# Combination of Klinefelter Syndrome and Acromegaly: A Rare Case Report

**DOI:** 10.1097/MD.0000000000003444

**Published:** 2016-04-29

**Authors:** Hongjuan Fang, Jian Xu, Huanwen Wu, Hong Fan, Liyong Zhong

**Affiliations:** From the Department of Endocrinology, Beijing Tiantan Hospital, Capital Medical University (HF, JX, HF, LZ); and Department of Pathology, Peking Union Medical College Hospital, Peking Union Medical College, Chinese Academy of Medical Sciences (CAMS) (HW), Beijing, China.

## Abstract

Klinefelter syndrome (KS) is the most common chromosomal aneuploidy in male population, which demonstrates an unusual association with acromegaly. We herein present a rare case involving the confirmation of KS 2 years after surgical treatment for acromegaly.

A 27-year-old man presented with an acromegalic appearance. Endocrinological examination revealed a high growth hormone (GH) concentration, low testosterone concentration, and high follicle-stimulating hormone and luteinizing hormone concentration. Brain imaging revealed a 9 × 6 × 7− mm sellar low-density nodule suggestive of a microadenoma. Trans-sphenoidal surgery was undertaken, and immunohistochemistry revealed GH positivity. Two years after surgery, the patient underwent examination for infertility. He presented with diminished pubic hair, and small and firm testes. Hormonal assay revealed hypergonadotrophic hypogonadism on the basis of decreased serum total testosterone (<0.2 ng/mL), and elevated luteinizing hormone (14.71 mIU/mL) and follicle-stimulating hormone (21.8 mIU/mL). A chromosomal karyotype examination showed 47,XXY, confirming the diagnosis of KS. Replacement therapy with oral testosterone undecanoate was begun. Brain imaging showed no delayed enhancement in the saddle region of the pituitary gland, but the concentration of plasma insulin-like growth factor maintained a high level. The patient's GH concentration was not significantly suppressed by the GH glucose suppression test. In this consideration, he was referred for postoperative somatostatin analogue treatment to control GH hypersecretion.

The misdiagnosis or delayed diagnosis of KS is mainly because of substantial variations in clinical presentation and insufficient professional awareness of the syndrome itself. As the simultaneous occurrence of KS and acromegaly is rare, and the association between them remains unclear, we suggest that complete pituitary hormonal screening and conventional pituitary MRI should be essential for patients with KS to screen for pituitary tumor.

## INTRODUCTION

Klinefelter syndrome (KS) is the most common form of hypogonadism and chromosome aneuploidy (0.15%) in the general male population.^1^ Owing to the paucity of significant manifestations, only 10% of patients with KS are diagnosed before puberty.^2^ The most common chromosomal karyotype is 47,XXY, which is present in about 80% of patients with KS.^3^ Three main clinical signs suggest the diagnosis of this disease in a child: small testes, tall stature, and mental retardation or learning problems.^4^

Acromegaly is a disorder characterized by growth hormone (GH) hypersecretion, multisystem-associated morbidities, and increased mortality. Most cases of acromegaly are because of a pituitary adenoma, which results in hypersecretion of GH and an elevated concentration of insulin-like growth factor 1 (IGF-1).^5^ To the best of our knowledge, the simultaneous occurrence of both KS and acromegaly has never been reported in the literature. We herein report a rare case of a double syndrome involving both KS (47,XXY) and acromegaly and describe the clinical features, treatment, and final outcome.

## CASE REPORT

**FIGURE 1:**
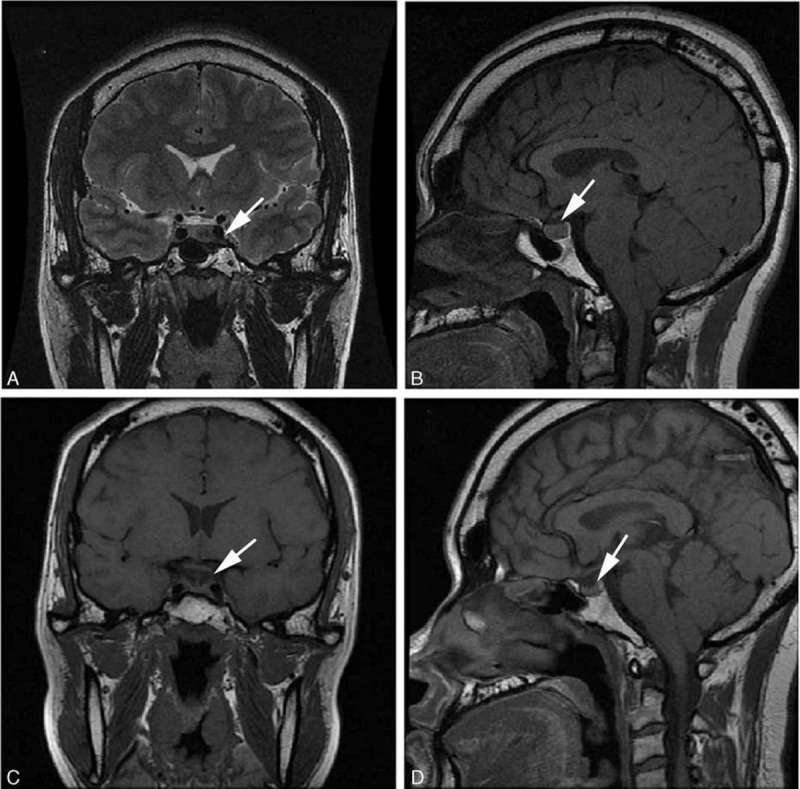
Head MRI. (A, B) A preoperative postcontrast coronal image shows a 9 × 7 × 6 mm slightly low-density shadow with an unclear border (white arrow). (C, D) After the operation, MRI showed no obvious abnormal signal in the lesion (white arrow).

In May 2013, a 27-year-old man was admitted to his local hospital for a physical examination because of a 5-year history of progressive hand enlargement and lip thickening. The patient developed typical features of acromegaly, including an enlarged head circumference, head skin thickening, mandibular prominence, prominent eyebrows and cheekbones, an increased nose size, tongue hypertrophy, widening of the feet (shoe size increased from 38 to 43), and hyperhidrosis. He reported no headache, visual field defects, or eye movement disorders. He had small, firm testes and impaired sex drive, but without other characteristic appearance of KS (ie, a tall, slender body with long legs and short torso, and development of breast tissue). The patient was 172-cm tall, weighed 94 kg, and had a body mass index of 31.8 kg/m^2^. Endocrinological examination revealed hypersecretion of GH, elevated luteinizing hormone (LH) and follicle-stimulating hormone (FSH) concentrations, and a low testosterone (T) concentration. His plasma adrenocorticotropic hormone, prolactin, and thyroid hormone concentrations were normal (Table 1). Head magnetic resonance imaging (MRI) showed an approximately 9 × 7 × 6 mm, slightly low-density shadow with an unclear border, suggestive of a microadenoma (Figure 1A, B).

**TABLE 1:**
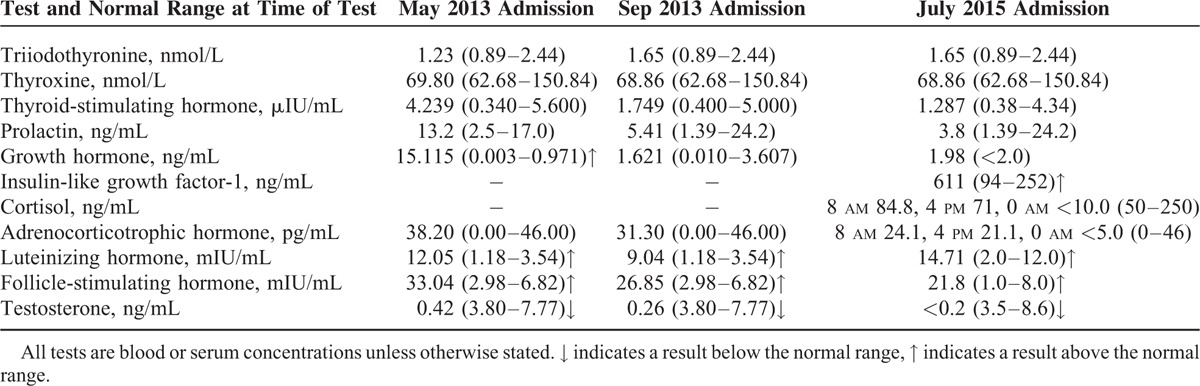
Endocrine Investigations Over Time

**FIGURE 2:**
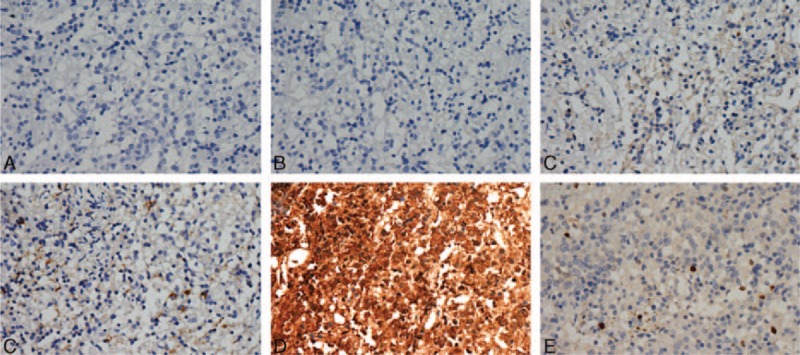
Immunohistochemistry of the pituitary microadenoma tissue sampled. (A) Negative for adrenocorticotrophic hormone. (B) No staining for follicle-stimulating hormone. (C) Weakly positive for luteinizing hormone. (D) Weakly positive for prolactin. (E) Strong staining for growth hormone. (F) Ki-67 proliferation index of approximately 2% (magnification ×200).

The patient underwent trans-sphenoidal surgery, and the mass was totally resected (Figure 1C, D). Immunohistochemical staining was negative for adrenocorticotropic hormone and FSH, weakly positive for LH and prolactin, and strongly positive for GH, and the Ki-67 proliferation index was approximately 2% (Figure 2A–E). Postoperative endocrinological examination 3 months later revealed that the serum GH concentration was 1.621 ng/mL (reference range, 0.010–3.607 ng/mL). The patient's symptoms had improved, including resolution of foot enlargement and hyperhidrosis.

**FIGURE 3:**
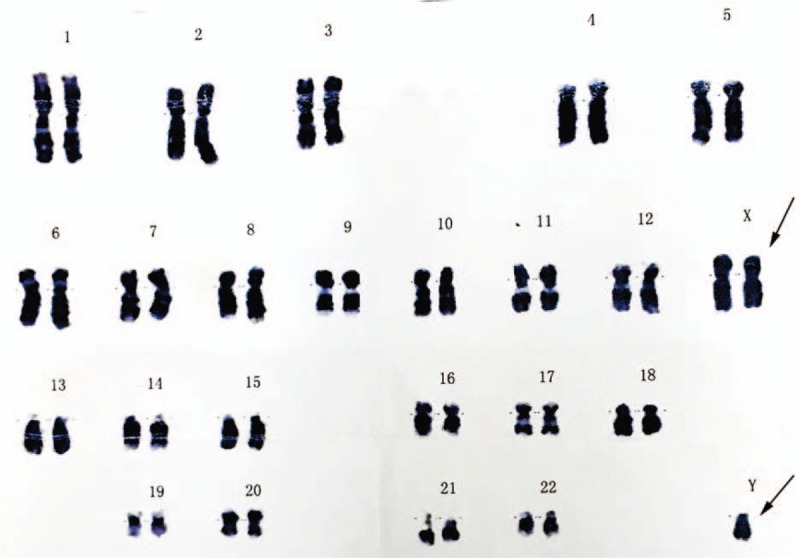
Chromosome karyotype examination showing 47,XXY. Chromosome karyotype shows total of 47 chromosomes, among them 2X and 1Y (black arrow).

In July 2015 (2 years after surgery), the patient came to our department for evaluation of infertility, after he had been married for 4 years. He exhibited acromegalic facies, was beardless and had scarce armpit hair growth, but was Tanner stage 3 for pubic hair development. His upper bust was 85 cm, under bust was 87 cm, and he had finger spacing of 172 cm. His penile length and circumference were 4 cm and 3 cm, respectively, and his testicular volume was small at about 2 mL each with solid quality. The patient reported a nearly normal erection and ejaculation, but was dissatisfied with the sexual desire and quality of the sexual life. The patient's hormone examination results are summarized in Table 1 and included low T (<0.2 ng/mL; reference range, 0–2 ng/mL), and high FSH (14.71 mIu/mL; reference range, 2–12 mIu/mL) and LH (21.8 mIu/mL; reference range, 1–8 mIu/mL) concentrations. Bone age testing indicated that the growth plates had closed. Sanger sequencing was used to search for point mutations and deletions in the short stature homeobox-containing gene (*SHOX*); however, no definite pathogenic mutations were found. Three sequential semen tests showed no sperm in his semen. Chromosome analysis revealed 47,XXY (Figure 3). KS was diagnosed and T replacement therapy was initiated.

**FIGURE 4:**
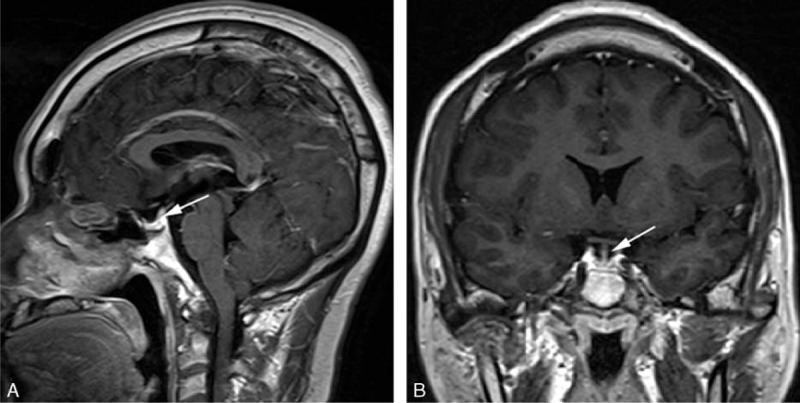
Head magnetic resonance imaging 2 years after surgery in July 2015: postcontrast coronal image shows no delayed enhancement in the saddle region (white arrow).

Head MRI showed no delayed enhancement in the saddle region (Figure 4), and endocrinological examination revealed a normal GH level at 1.98 ng/mL (reference range, 0.0–2.0 ng/mL) and a high IGF-1 level for his age and sex (Table 1). Plasma GH and blood glucose concentrations were measured 0, 30, 60, 90, 120, and 180 minutes after administration of 75 g of oral glucose. The most broadly accepted definition of biochemical “cure” of acromegaly resulting from the American Association of Clinical Endocrinologists (AACE) is normalization of the basal GH level <2.5 mg/L, suppression of GH to 1 mg/L during the oral glucose tolerance test, and normal IGF-I for age and sex.^6^ The GH nadir was >1 ng/mL during a 75 g oral glucose tolerance test and the elevated IGF-1 level for the patient's age and sex, which indicates incomplete remission (Table 2) and necessitated further medical therapy. After treatment with 0.1 g of subcutaneous Sandostatin (octreotide acetate, Novartis Pharmaceuticals Corporation) every 8 hours for 3 days, the patient's GH and IGF-1 concentrations were 0.81 and 522 ng/mL, respectively. Somatostatin analogues have been proven to be effective in the hormonal control of our patient; thus, he was treated with 30 mg of Somatuline LA (lanreotide, Novartis Pharmaceuticals Corporation) by intramuscular injection to control the GH concentration once every 14 days after discharge.

**TABLE 2:**
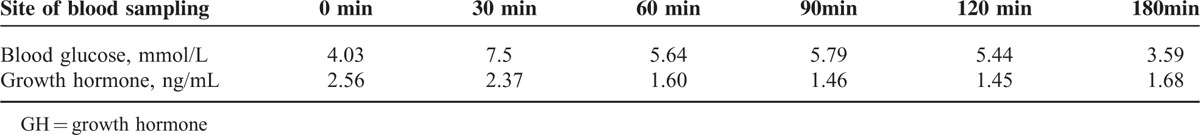
Serum GH with Oral Glucose Tolerance Test

## DISCUSSION

This case describes the presence of a GH-secreting pituitary microadenoma in a patient with KS, which was previously undiagnosed. Early recognition and hormonal treatment of KS can substantially improve patients’ quality of life and prevent serious consequences.^7^ The low rate of timely discovery and diagnosis results from diversity in clinical presentation of the patient and insufficient understanding of the classic endocrine negative feedback system by physicians. More emphasis should be placed on improving case identification of KS. We diagnosed him with KS based on his atrophic testicles, primary hypogonadism as revealed by hormonal examination, and a chromosomal aberration of 47,XXY.

In male individuals, hypogonadism can be divided into 2 types: hypogonadotropic hypogonadism owing to a lesion in the hypothalamic or pituitary region and hypergonadotropic hypogonadism from defects in the testes or in the androgen target tissues.^8^ The tumor mass effects and invasive alternative, pituitary hormone decline was frequently observed in the anterior lobe of the pituitary gland before and after trans-sphenoidal surgery, which leads to hypogonadotropic hypogonadism.^9^ Hypergonadotropic hypogonadism can result from testicular injury, tumour, or infection and genetic defects affecting testicular development (eg, KS), as well as chemotherapy, radiation treatment or alcohol abuse.^8^ KS is the most common genetic form of hypergonadotropic hypogonadism. According to patient's history and clinical examination, KS was given higher priority. For our patient, a low T concentration with elevated FSH and LH concentrations in this case provided a clue to KS diagnosis, and the confirmed diagnosis based on chromosome analysis.

The patient had typical features of acromegaly, and laboratory examination reveals a notable increase in the GH concentration. Furthermore, the imaging detected a pituitary adenoma and postoperative immunohistochemical findings confirmed the diagnosis of a pituitary GH-secreting microadenoma. Kontogeorgos et al^10^ defined pituitary gonadotropin-secreting adenomas as those with an FSH and/or LH expression level of >10%. In our patient, immunohistochemical staining was negative for FSH and weakly positive for LH. Gonadotropinoma or acromegaly combined gonadotropinoma should be excluded.

Acromegaly is a rare disease with unrestricted GH-induced secretion of IGF-1, leading to an increased prevalence of comorbidities.^11^ Treatment options include surgery, medical therapy, and radiotherapy. Trans-sphenoidal surgery is recommended as a priority treatment for many patients with acromegaly, particularly those with microadenomas.^12^ However, 40% to 60% of patients with acromegaly will experience persistent or recurrent disease following surgery, necessitating additional therapy.^13^ Somatostatin analogues are the first-line medical treatment for patients with acromegaly. Moreover, better prognosis of acromegaly depends upon early recognition, diagnosis, and management.^5^ A multidisciplinary management approach and close follow-up are thus necessary for acromegaly therapy.^14^

The patient's skin was black and rough, and his height was normal, which led to diagnostic confusion and resulted in misdiagnosis during his first treatment. His parents were normal in appearance, intelligence, and height (father, 170 cm; mother, 156 cm). The patient's mother stated that the development of the patient's language and motor ability was delayed and that his height growth was slow. As he reached maturity, the patient's language and motor ability became almost normal. At the age of 22 years, he was 160-cm tall and continued growing until he reached 172 cm. He had been a manual worker in an industrial company after his graduation from junior high school. He had a mild temperament with no violent behavior. He got married at the age of 25, and because of low sexual desire, he had received testosterone treatment for half a year.

The *SHOX* gene on chromosome X influences the phenotype of KS; the gene is situated in the pseudoautosomal region 1 on Xp. *SHOX* hapo insufficiency has been implicated in growth retardation and bone changes in Turner syndrome^15^ and Leri–Weill^16^ dyschondrosteosis and is also implicated in the slightly accelerated growth in KS, 47,XXX and 47,XYY syndrome.^17^ Overexpression of the *SHOX* gene is likely to be responsible for the normal stature seen in patients with KS.^18,19^ No definite pathogenic mutation was found in the patient's SHOX sequence.

A characteristic of KS is tall stature, but not all men with XXY actually develop this syndrome or its symptoms. Many autosomal genes are differentially expressed in patients with KS, which explains the diversity in clinical phenotype diversity among patients with KS.^20,21^ In fact, many men show no abnormalities at all. Some uncommon KS variants are associated with short stature (49,XXXXY).^22^ There are a few reported cases of KS and short stature secondary to GH deficiency.^23–31^ Complementary studies showed partial GH deficiency, but the reason for such an association is not clearly known.

We believe that the patient's height increase was a result of his GH-secreting pituitary adenoma, and the possibility that this patient with KS had short stature because of partial GH deficiency cannot be entirely ruled out. KS can stimulate tallness because of failure of the epiphyses to close. The gonadal steroids (estrogen, T) cause closure of the epiphyseal growth plates.^32^ On this basis, we hypothesized that the T treatment for low libido that the patient received after marriage might account for the closure of the epiphyses.

Prolonged target gland failure causes pituitary hyperplasia; several cases of gonadotrope cell hyperplasia were reported in patients with KS.^33^ Samaan et al^34^ have reported that reactive pituitary changes may accompany the gonadal failure present in these dysgenetic syndromes. It is reasonable to assume that because of the loss of negative feedback, chronic stimulation of adenohypophysial cells type results in hyperplasia.^35^ Whether hyperplastic gonadotroph cells can transform to adenoma and the underlying mechanism is still not clear.

KS is characterized by at least 1 supernumerary X chromosome in addition to the genes on the extra X chromosome, which may be inherited from either parent.^36^ Acromegaly is an acquired disorder related to excessive production of GH and characterized by progressive somatic disfigurement and systemic manifestations.^37^ The *gsp* gene mutation is the major intrinsic defect in the pathogenesis of acromegaly.^38^ In addition, acromegaly in the setting of germline *AIP* mutations has been reported in clinical studies worldwide.^39,40^ So we believe that the KS occurred before the acromegaly, and whether the formation of GH-secreting pituitary adenoma is because of a genetic abnormality linked to KS remains to be elucidated.

## CONCLUSION

Timely diagnosis of KS is challenging itself, and the presence of a GH-secreting pituitary adenoma in this case suggests a rare combination. Further reports would be needed to confirm a possible association between KS and acromegaly. Better understanding of the classic endocrine negative feedback system is important for early diagnosis in KS. Our report also highlights the importance of complete pituitary hormonal screening and conventional pituitary MRI might be essential for the patients with KS to screen for the presence of pituitary adenoma.
